# Antibiotic therapy for snakebite envenoming

**DOI:** 10.1590/1678-9199-JVATITD-2019-0098

**Published:** 2020-02-03

**Authors:** Dabor Resiere, José María Gutiérrez, Rémi Névière, André Cabié, Mehdaoui Hossein, Hatem Kallel

**Affiliations:** 1 Intensive Care Unit, University Hospital of Martinique, Fort-de-France, Martinique.; 2 Instituto Clodomiro Picado, Facultad de Microbiología, Universidad de Costa Rica, San José, Costa Rica.; 3 Department of Infectious Diseases, University Hospital of Martinique, Fort-de-France, Martinique.; 4 Intensive Care Unit, Cayenne General Hospital, Cayenne, French Guiana.

## Abstract

There are numerous conflicting recommendations available on the use of antibiotics following snakebite. The present letter to the editor presents some recommendations based on recent studies, and aims to stimulate debate on this topic.

Dear Editor, 

According to international guidelines amoxicillin-clavulanate is recommended to prevent secondary infections from animal bites [1]. However, the systematic antibiotic administration after snakebite is questionable. Most authors recommend antibiotics for severely bitten patients or when inflammatory signs are suggestive of infection. Interestingly, empiric amoxicillin-clavulanate use was shown to be ineffective in preventing secondary infections after snakebites, because of the resistance to β-lactam antibiotics in the bacterial species commonly found infecting snakebite site [2]. Recently, analysis of the antibiotic susceptibility of bacteria isolated from *Bothrops lanceolatus* mouth showed 67% of strains resistant to amoxicillin-clavulanate, whereas the majority of isolated bacteria were susceptible to third-generation cephalosporins [3].

Wound infection following snakebite usually accounts for 9 to 77% of the bitten patients, as described in several studies [2, 4-8]. The large differences in the reported prevalence of secondary infections in snakebites can be related to variations in the criteria used to establish the presence of infection. Indeed, there is no precise set of clinical criteria to define infection in snakebite envenomings. In addition, a high proportion of microbiological cultures are negative because of systematic preemptive use of antibiotics in snake bitten patients. The main involved bacteria are *Enterococcus faecalis*, *Aeromonas hydrophila* and *Morganella morganii* [2, 4-9].

The snake mouth is colonized by bacteria that can be transmitted to the bitten patient through the skin injury associated with the bite [3, 10-12]. Inoculation of bacteria from the mouth, fangs, or venom following snakebite can cause local infection with abscess and necrotizing fasciitis in most severe cases [13]. In one recent study, isolated Enterobacteriaceae following snake bite infection showed 69% resistance to ampicillin, 60% resistance to amoxicillin/clavulanate, and 66% resistance to second-generation cephalosporins [13]. Conformingly, our experimental study examining the bacteria sampled from the oral cavity of 26 *B. lanceolatus* specimens collected from various areas in Martinique supported that 67% of the isolated bacteria were resistant to amoxicillin/clavulanate. In addition, the majority of isolated bacteria were susceptible to third-generation cephalosporins (i.e., 73% to cefotaxime and 80% to ceftazidime) [3]. Based on the most frequently isolated bacteria and susceptibility profiles documented in cases of infection after snakebite, active antibiotics include third generation cephalosporins, piperacillin-tazobactam and ciprofloxacin.

In conclusion, preemptive antibiotic administration in snake-bitten patients should be considered only in those with severe local signs of envenomation, and empiric one in those having local or general signs of infection, regardless of the degree of envenoming. The most appropriate empirical antibiotics are third generation cephalosporins. Empirical amoxicillin-clavulanate should no longer be used in this context.
